# Detecting Substance Use Disorder Using Social Media Data and the Dark Web: Time- and Knowledge-Aware Study

**DOI:** 10.2196/48519

**Published:** 2024-05-01

**Authors:** Usha Lokala, Orchid Chetia Phukan, Triyasha Ghosh Dastidar, Francois Lamy, Raminta Daniulaityte, Amit Sheth

**Affiliations:** 1 Department of Computer Science and Computer Engineering, Artificial Intelligence Institute, University of South Carolina, Columbia, SC, United States; 2 Department of Computer Science and Engineering, Indraprastha Institute of Information Technology, Delhi, India; 3 Department of Computer Science and Engineering, Birla Institute of Technology & Science Pilani, Hyderabad, India; 4 Department of Society and Health, Mahildol University, Salaya, Thailand; 5 College of Health Solutions, Institute for Social Science Research, Arizona State University, Phoneix, AZ, United States

**Keywords:** opioid, substance use, substance use disorder, social media, US, opioid crisis, mental health, substance misuse, crypto, dark web, users, user perception, fentanyl, synthetic opioids, United States

## Abstract

**Background:**

Opioid and substance misuse has become a widespread problem in the United States, leading to the “opioid crisis.” The relationship between substance misuse and mental health has been extensively studied, with one possible relationship being that substance misuse causes poor mental health. However, the lack of evidence on the relationship has resulted in opioids being largely inaccessible through legal means.

**Objectives:**

This study aims to analyze social media posts related to substance use and opioids being sold through cryptomarket listings. The study aims to use state-of-the-art deep learning models to generate sentiment and emotion from social media posts to understand users’ perceptions of social media. The study also aims to investigate questions such as which synthetic opioids people are optimistic, neutral, or negative about; what kind of drugs induced fear and sorrow; what kind of drugs people love or are thankful about; which drugs people think negatively about; and which opioids cause little to no sentimental reaction.

**Methods:**

The study used the drug abuse ontology and state-of-the-art deep learning models, including knowledge-aware Bidirectional Encoder Representations From Transformers–based models, to generate sentiment and emotion from social media posts related to substance use and opioids being sold through cryptomarket listings. The study crawled cryptomarket data and extracted posts for fentanyl, fentanyl analogs, and other novel synthetic opioids. The study performed topic analysis associated with the generated sentiments and emotions to understand which topics correlate with people’s responses to various drugs. Additionally, the study analyzed time-aware neural models built on these features while considering historical sentiment and emotional activity of posts related to a drug.

**Results:**

The study found that the most effective model performed well (statistically significant, with a macro–*F*_1_-score of 82.12 and recall of 83.58) in identifying substance use disorder. The study also found that there were varying levels of sentiment and emotion associated with different synthetic opioids, with some drugs eliciting more positive or negative responses than others. The study identified topics that correlated with people’s responses to various drugs, such as pain relief, addiction, and withdrawal symptoms.

**Conclusions:**

The study provides insight into users’ perceptions of synthetic opioids based on sentiment and emotion expressed in social media posts. The study’s findings can be used to inform interventions and policies aimed at reducing substance misuse and addressing the opioid crisis. The study demonstrates the potential of deep learning models for analyzing social media data to gain insights into public health issues.

## Introduction

### Background

North America is facing the worst opioid epidemic in its history. This epidemic started with the mass diversion of pharmaceutical opioids (eg, oxycodone, hydromorphone), resulting from the strong marketing advocacy of the potential benefits of opioids [1]. The increase in opioid use disorder prevalence and pharmaceutical opioid-related overdose deaths resulted in a stricter distribution of pharmaceutical opioids, unintentionally leading to a substantial increase in heroin use among pharmaceutical opioid users [2]. The epidemic entered its third wave when novel synthetic opioids (eg, fentanyl, U-47700, carfentanil) emerged on the drug market. Recent research and several reports are pointing at the role of cryptomarkets in distributing novel psychoactive substances [3,4]. The importance of cryptomarkets has been further exacerbated by the spillover mental health and anxiety resulting from the ongoing COVID-19 pandemic: recent results from the Global Drug Survey suggest that the percentage of participants who have been purchasing drugs through cryptomarkets has tripled since 2014, reaching 15% of the 2020 respondents [5]. In this study, we assess social media data from active opioid users to understand the behaviors associated with opioid use and to identify what types of feelings are expressed. Substance use disorder (SUD) in social media posts is defined as a post that shows the risk of substance use, attitudes, and behavior related to substance use, as well as the corresponding social and environmental factors [6]. We used deep learning models to perform sentiment and emotion analysis of social media data with the drug entities derived from cryptomarkets. We implemented state-of-the-art sentiment and emotion models for social media data. Additionally, we performed topic analysis to extract frequently discussed opioid-related topics in social media. For the preliminary analysis, we examined temporal variations in topics that differentiate between posts at each drug level and topics over time across all years, followed by considering data per quarter for each year. We also analyzed how users’ language in their posts varies temporally by topic. We also observed variations in emotions and sentiment that differentiate between posts containing expressions of SUD. For this task, we fine-tuned a pretrained transformer language model for emotions and sentiments, used it to automatically extract the emotions and sentiments for all historical posts related to a drug, and analyzed variations in sentiment and emotion over time. We further aim to achieve the identification of SUD on social media by examining the core research question of this study: can we differentiate between posts containing expressions of substance misuse or not with temporal activity, emotion, sentiment, and language features related to that drug? We built a knowledge-aware bidirectional sequential neural model that differentiates between posts where expressions of SUD are present versus those posts where it is absent.

### Findings and Contributions

The major contributions and findings of this work are as follows:

- We compiled a high-quality, rare, challenging, and valuable dark web data set (eDark) by crawling four cryptomarkets, namely, Dream, Tochka, Agora, and Wall Street. The data set is available for release upon acceptance.
- We propose an end-to-end architecture (dark web to social media) for harnessing social media trends for opioid listings found on the cryptomarket. It involves crawling techniques, drug identification, data collection, processing from social media, and computational models to predict SUD considering the temporal variations in sentiment and emotional language among posts indicative of SUD. We also contribute to the knowledge- and historic posts–aware sequential neural model that can differentiate if SUD is present or absent for a drug based on these variations by factoring in the relative time difference between historical posts. We present that knowledge-, sentiment-, and emotion-aware models outperform other models of language feature–based approaches by performance measures, ablation study, and error analysis.
- To the best of our knowledge, our work is the first one to detect SUD in social media posts considering the above factors and as a reflection of the opioid listings extracted from the dark web. Resources created as a part of the study will be made available upon request to the corresponding author upon acceptance. The resources include emotion-, sentiment-, and SUD-labeled data sets with timestamps for each drug type, and the eDark data set.

### Related Work

#### Dark Web Marketplaces

The dark web serves as a favorable and promising market for illegitimate goods ranging from drugs to weapons [7-9]. ElBahrawy et al [10] investigated the market dynamics of dark web markets based on a unique data set of Bitcoin transactions. They have also analyzed how the market ecology restructures itself once it closes. As traditional web scraping tools have failed to remove the veil of the vendors of dark marketplaces, Hayes et al [11] proposed an automated framework to overcome this barrier. The suggested framework was further evaluated by gathering information from 3000 sellers on a dark marketplace. Harviainen et al [12] presented an analysis of the pattern that the buyers and the sellers expose themselves on Sipulitori (a Finnish dark web drug trading market). Hassio et al [13] extended research on Sipulitori by exploring it from the viewpoint of understanding the needs behind the messages posted by users and the physiological and cognitive factors that come into play. Researchers examined the underground marketplaces Agora and Dream Market to examine fluctuations in the availability of fentanyl, fentanyl analogs, and other illegal opioids in connection to overdose fatalities [14]. Orsolini et al [15] provided intuition behind dark web drug marketplaces through the perspective of psychiatrists so that they can be equipped with adequate information for providing countermeasures to increasing addictions to drugs available through these marketplaces. The prior work on analyzing dark web marketplaces suggests that such data could detect trends in the real world. Next, we discuss how time series analysis on social media helps to quantify such trends.

#### Time Series Analysis on Social Media

Earlier research has demonstrated the use of time series analysis on social media data, such as for comprehending changes in the sentiment of the public’s perceptions, which can be beneficial to the government and commercial organizations [16], and understanding the sentiments of users of addictive smartphone apps such as PUBG and TikTok [17]. Time series analysis has also been used in research on mental health [18], such as variations in individuals’ mental health throughout the COVID-19 lockdown phase [19]. A study conducted among adolescents in Nigeria examined how peer pressure and substance use affect the mental health of in-school adolescents, finding significant associations between these factors [20]. It highlights the need for interventions addressing peer pressure and substance use to promote positive mental health outcomes among adolescents. Over time, topic analysis and sentiment analysis have been used to deepen the understanding of web-based retail customer behavior from tweets [21]. Researchers have used time series analysis to analyze bursts of activity in social networks, and for prediction, they have used a long short-term memory (LSTM) network–based model [22]. A sparse additive generative model, a topic analysis tool, was used to assess the temporal linguistic changes in tweets with and without evidence of self-harm. Furthermore, they explored temporal linguistic features of tweets with and without suicidal intent signs [23]. A transformer-based model was also proposed for suicidal ideation detection in social media that takes into consideration the temporal context [24].

#### Substance Use Analysis on Social Media

Several researchers have explored social media analysis for different investigations of drug use. These works have analyzed the content, sentiment, and emotion for drug-related data collected from social media platforms like Twitter and Instagram. Lossio-Ventura and Bian [25] worked on a large amount of opioid-related data collected from Twitter to gain an overall understanding of drug-related discussions on the platform, behavior related to drug consumption, drugs co-used, and street terms for various drugs. This study restated that Twitter had a huge corpus of data and could provide insights into its correlation with pain management and alcohol consumption. A similar study by Cherian et al [26] was conducted on Instagram data on the misuse of codeine. The temporal data collected related to codeine misuse showed its interconnection with alcohol and soda consumption. The influence of social media in propagating this imagery increases the risk of normalizing drug use to extremes. Kim et al [27] further explored how big data can be used to understand drug use and addiction better. Social media is a large platform for monitoring prescription drug use and addiction using linguistic and behavioral cues. The work done by Lokala et al [14] investigates the relation between the availability of fentanyl-related drugs on cryptomarkets on the dark web and overdoses of fentanyl. Time-lagged correlation analysis was done between fentanyl-related drugs from the cryptomarket and overdoses of fentanyl in this first-of-its-kind study for epidemiological surveillance. Sarker et al [28] investigated various opioid-related subreddits to better understand the differences in conversations concerning prescription/illegal opioids and access to SUD treatment during the pre–COVID-19 and COVID-19 periods. They also noticed a rise in opioid withdrawal discussions during COVID-19. Posts from various subreddits related to opioids (both medical and illicit) were collected to identify the increase in the use of stimulants among opioid users and individuals with opioid use disorder [29]. This corresponds to the increasing number of casualties because of opioid and stimulant overdoses. Desrosiers et al [30] reported the perseverance of negative sentiments in the conversations of individuals with increased drug use severity. Liu et al [31] outlined the presence of positive emotion in the Facebook posts of individuals who underwent SUD treatment for a longer period than those who stopped their therapy. A study was also conducted by Singh and Wu [32] to probe the sentiment patterns of tweets related to SUD before and during the COVID-19 pandemic. Cameron et al [33] followed the development of a semantic web platform called Prescription Drug Abuse Online Surveillance and Epidemiology (PREDOSE) for harvesting data related to prescription drug use from social media platforms. Supporting several types of content analysis, PREDOSE provides easy access to data for drug use research. Fan et al [34] illustrated a new framework called AutoDOA to detect drug addiction behavior from Twitter. This will aid in understanding patterns of drug use and addiction. Eshleman et al [35] discussed how social media can be leveraged for drug recovery. Using linguistic patterns and machine learning algorithms, identifying groups of people more likely to participate in the drug recovery process would be an important step in managing the drug addiction epidemic. Our work aims to build an end-to-end system where we can see the reflection of the dark web on social media in terms of trends, sentiment, emotion, and substance use context, which is necessary for timely public health interventions.

### Data Collection

**Figure 1.**
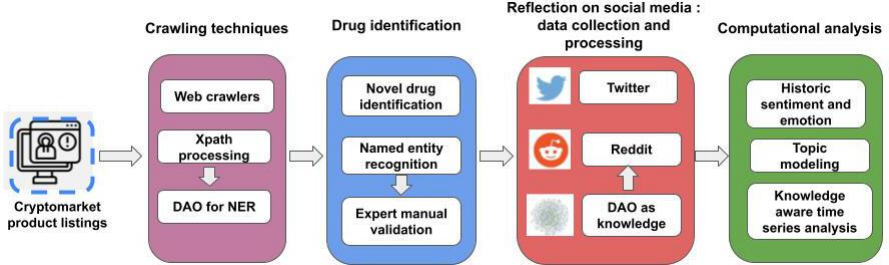
Proposed dark web to social media architecture for harnessing social media trends for listings found on the cryptomarket eDark data collection. DAO: drug abuse ontology; NER: named-entity recognition.

This section presents the modules-crawling techniques, drug identification, and data collection proposed in the dark web to social media architecture as shown in Figure 1.

Concerning dark web data, four cryptomarkets, Agora, Dream Market, Tochka, and Wall Street, were periodically crawled between June 2014 and January 2020. Over 82,000 opioid-related listings were collected to extract posts about fentanyl, fentanyl analogs, and other nonpharmaceutical synthetic opioids in the cryptomarket. The data sources include four different cryptomarkets. Further, we discuss the eDark data set summary and a description of cryptomarkets in this section.

### Dark Web Data (eDark) Collection and Summary

#### Dream Market

The marketplace was established in late 2013. Dream Market, after AlphaBay, was the largest dark web market in the world before 2017. Nevertheless, Dream Market quickly overtook AlphaBay as the largest dark web market in the world once AlphaBay went down in 2017 [36]. Between November 2014 and April 2019, there were 261 withdrawals from the market in total. During this time, the market saw transactions worth over US $197,000 [37].

#### Tochka

The marketplace started operating in 2015. It is a fairly modest market that mostly operates in North America and Europe. More than 3621 listings, including pharmaceuticals, malware, and other products, are sold on the website. The market changed its name to the Point Market and is currently open [38]. Between November 2014 and April 2019, there were a total of 2990 withdrawals from the market.

#### Wall Street

The marketplace features a site for the sale of illegal substances, weapons, hacking tools, and stolen log-in information. However, the exit scam has been hurting the market since April 2019 [39]. The administrators allegedly stole between US $30 million worth of XMR and Bitcoins from vendor accounts by switching the site into maintenance mode and transferring the clients’ funds [40]. In May 2019, the market was later shut down. Before being taken over in May 2019 by the German Federal Criminal Police, Wall Street was the second-largest dark web market in the world. In total, 7755 withdrawals were made from the market between November 2014 and April 2019. During this time, there was almost US $18,000 worth of transactions on the market [37].

#### Agora

The marketplace was a dark web market in operation from September 2013 to August 2015 that sold illegal narcotics and controlled substances, drugs, counterfeit and fraud-related goods, services, and other illegal contraband. The data for Agora from June 2014 to September 2015 was obtained from the Grams data set [9]. Agora was chosen because it was one of the largest cryptomarkets that emerged after the Federal Bureau of Investigation shut down of Silk Road [41].

#### Data Summary

The summary of the dark web data set is shown in Table 1.

**Figure 2.**
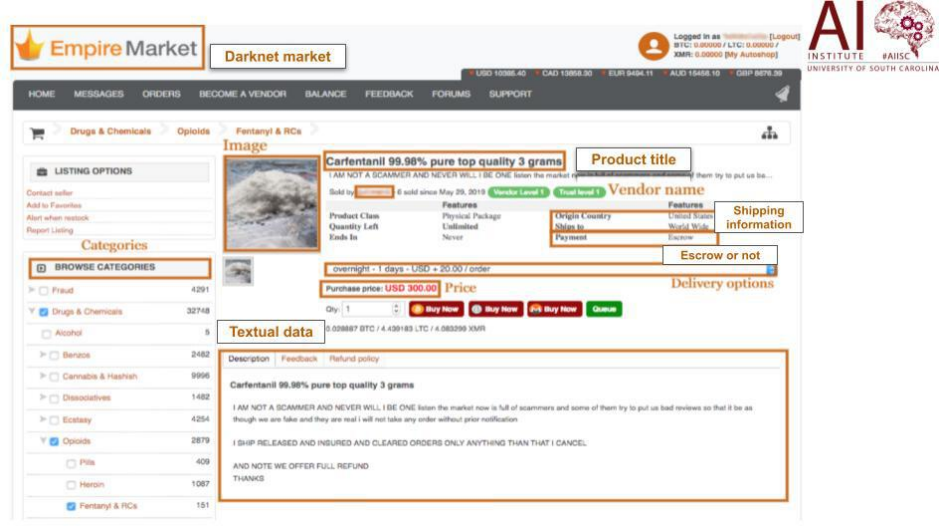
Data source of eDark: a sample product listing page from the Dream Market cryptomarket.

A sample product page of Dream Market is shown in Figure 2. The Scrapy framework was used to create the unique web crawler for each market, circumventing security protections built into these markets. To get over security safeguards, the web crawler uses specialized Scrapy downloader middleware. By creating a Linux virtual machine on Amazon Web Services running the Tor daemon and Privoxy, the custom crawler was able to reach the deep web. The outputs of the crawler are unaltered HTML files used for drug advertising. The university’s information security office evaluated and approved the data extraction, storage, and access processes, which all adhered to stringent security standards. The information that was extracted from the data included the following: the product name provided by the vendor, the vendor screen pseudonym, the number of sales made by the vendor and their level of trust, the drug names, drug category, the information the vendor provided about the product, the unit, the quantity in stock, the price (in Bitcoin and US dollars), the price per volume, the country/region of origin, the destination country/region, and the security precautions for transactions. We further used a custom-built named-entity recognition (NER) algorithm to extract substance names, product weight, price of the product, shipment information, availability, and administration route as shown in Table 2. The NER algorithm consists of three key components: (1) the Natural Language Toolkit is used to curate and process text portions from crawled data; (2) the drug abuse ontology (DAO) that serves as a conceptual framework for interconnecting groups of drug-focused lexicons to produce a list of items to be identified; and (3) regular expressions, which are a sequence of symbols and characters that create a pattern that can be searched in text or a sentence constructed using the DAO-selected entities to extract things of interest.

**Table 1.** eDark summary.

Data	Dream Market, n	Tochka, n	Wall Street, n	Agora, n
Vendors	3456	765	876	910
Substances	2862	679	765	821
Locations	436	62	37	214
Dollar worth (US $)^a^	197,000	5072	18,000	220,000
Withdrawals^a^	262	2990	7755	844

a US dollar values and withdrawals are approximated to the nearest value.

**Table 2.** Sample of property types in eDark identified from cryptomarket product listing.

Property name	Cryptomarket listing information
Has product name	50 Gr ***** Heroin AAA +With Spots Free Shipping
Is substance	Heroin
Has class	Opiate
Has dosage	1.5 gram
Has quantity	50 gram
Has vendor	BulkBrigade
Has price	BTC 0.0444
Ships to	Worldwide
Ships from	Germany

### Named-Entity Recognition

Extracted data included features like product name, vendor screen name (vendor name), drug category, product description, price (Bitcoin or US dollar), country/region of origin and destination, how to administer the drug, shipping information, and others. We used a pretrained NER deep learning bidirectional LSTM–convolutional neural network (CNN) approach [42] on cryptomarket data to identify drug entities that use a hybrid bidirectional LSTM and CNN architecture, eliminating the need for most feature engineering. The entities are then matched to a superclass using DAO [43] that acts as a domain-specific resource. DAO is a domain-specific knowledge source containing drug- and health-related classes, properties, relationships, and instances. Apart from medical terms, it includes concepts of mental health disorders and symptoms aligned with the *Diagnostic and Statistical Manual of Mental Disorders* (Fifth Edition) scale. DAO is a domain-specific conceptual framework for interconnecting sets (named “classes”) of drug-focused lexicons. One of the key benefits of using an ontology-enhanced semantic approach is the ability to identify all variants of a concept in data (eg, generic names, slang terms, scientific names). The DAO contains names of psychoactive substances (eg, heroin, fentanyl), including synthetic substances (eg, U-47700, MT45), brand and generic names of pharmaceutical drugs (eg, Duragesic, fentanyl transdermal system), and slang terms (eg, roxy, fent). It also contains information regarding the route of administration (eg, oral, intravenous), unit of dosage (eg, gr, gram, pint, tablets), physiological effects (eg, dysphoria, vomiting), and substance form (eg, powder, liquid, hydrogen chloride). Initially, it was used to determine user knowledge, attitudes, and behaviors related to the nonmedical use of buprenorphine and other illicit opioids through analysis of web forum data. Later, this ontology evolved to understand trends of drug use in the context of changing legalization policies in the United States. This also proved effective in capturing gleaning trends in the availability of novel synthetic opioids through the analysis of cryptomarket data. DAO is defined as using a common ontology methodology known as 101 ontology development. The 101 technique entails the following steps: (1) establishing the ontology’s domain and scope, (2) reusing prior knowledge, (3) enumerating key terms in the ontology, (4) defining classes and their properties, and (5) producing instances of the classes. A collection of techniques and best practices accepted by the Semantic Web community and the artificial intelligence community that perform natural language processing were used to assess the ontology’s quality. Protege is the most used tool for creating ontologies [44], so the metrics list the numbers for its structures and representation. The DAO metrics were evaluated as shown in Table 3.

**Figure 3.**
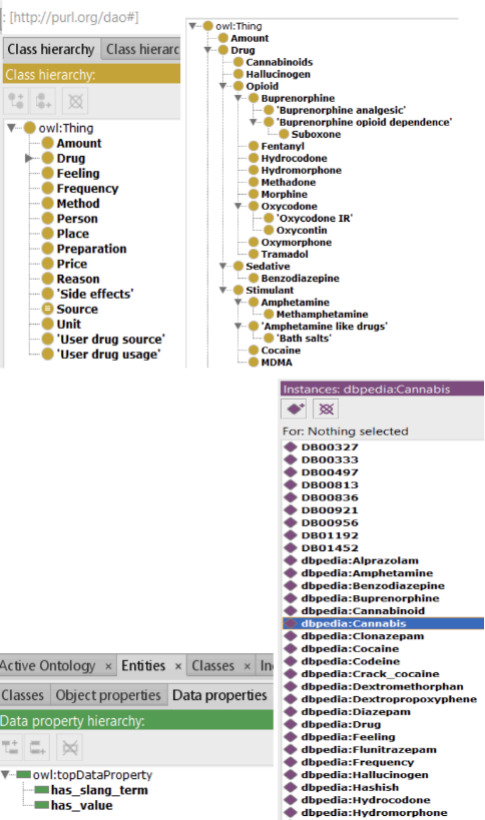
The Web Ontology Language representation of drug abuse ontology.

The Web Ontology Language representation of DAO is presented in Figure 3. In this study, we leveraged DAO to identify 90 drug entities, which we then broadly classified into 8 categories by mapping each entity to a super drug class in DAO. The 8 broad categories considered were heroin, synthetic heroin, pharmaceutical fentanyl, nonpharmaceutical fentanyl, fentanyl, oxycodone, kratom, and opium (chosen as per data availablity in each category on social media). The categorization of the 5 types of opioid listings containing specific types and subclasses identified using DAO is shown in Textbox 1.

**Table 3.** Drug abuse ontology metrics.

Ontology metric	Value, n	Description
Axiom	4876	Number of combined logical and nonlogical axioms
Logical axiom count	3478	Number of logical axioms
Declaration axiom count	1185	Number of declaration axioms
Classes	316	Number of distinct classes
Objects	12	Number of object properties
Data property	13	Number of data properties
Individual count	845	Number of individual entities

Textbox 1. **Opioid listings categories, subclasses, and specific types identified using drug abuse ontology.**

**Pharmaceutical fentanyl**

Duragesic, Sublimaze, fentanyl transdermal system

**Nonpharmaceutical fentanyl**

Oxycodone pills with fentanyl

**Fentanyl analogs**

Acetylfentanyl, acrylfentanyl, alfentanyl, benzylfentanyl, betahydroxyfentanyl, betamethylfentanyl, butryfentanyl, carfentanil, crotonylfentanyl, etorphine, etorphinecartanil, fluorofentanyl, isobutyrfentanyl, lofentanyl, methoxyacetylfentanyl, methylfentanyl, etc

**Novel synthetic opioids**

U-50488, U-47700, U-49900, U-48800, MT-45, AH-7921, W-18, MPF-47700, etc

**Pharmaceutical opioids**

Buprenorphine, codeine, hydrocodone, hydromorphone, loperamide, methadone, morphine, naloxone, oxycodone, oxymorphone, tramadol

### Identifying Substance Use Discussions on Social Media

We crawled the data using a carefully curated lexicon extracted from DAO consisting of around 120 terms (slang names, brand names, drug names, street names, marketing names, commonly used names, and abbreviations) of those 8 drug categories.

**Figure 4.**
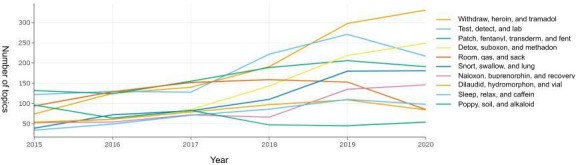
Dark web to social media topic modeling: module topics over time for all 8 drug categories from 2015 to 2020.

Using the compiled list, we collected 290,458 opioid-related posts from 6 subreddits using custom-built crawlers, which we call the Substance Use Disorder Corpus (SUDS). The 6 subreddits chosen for data collection were r/drug nerds, r/research chemicals, r/opiates, r/heroin, r/suboxone, and r/opiates recovery. The subreddit corpus is spread over different drug categories such as heroin (n=136,745), kratom (n=77,443), fentanyl (n=36,166), oxycodone (n=25,890), opium (n=9675), nonpharmaceutical fentanyl (n=2798), pharmaceutical fentanyl (n=876), and synthetic heroin (n=865). To build the social media emotion analysis model, additionally, we collected 151,563 posts from Twitter using the Twitter application programming interface with the same lexicon we used for the subreddit crawler. We applied term frequency–inverse document frequency (TF-IDF) over unigrams, bigrams, and trigrams to identify topics in each subreddit as shown in Textbox 2. We also conducted the topic analysis using the BERTopic [45] model for all drugs over time from 2015 to 2020, as shown in Figure 4.

Textbox 2. **Sample of topics identified from the Substance Use Disorder Corpus data set obtained from 6 different subreddits.**

**Opiates recovery**

Cold turkey withdrawal, cravings, anxiety, rehab, depression, sobriety, loperamide, benzo, Subutex, quitting, Vivitrol, Imodium, naltrexone

**Opiates**

Codeine, hydrocodone, oxymorphone, Dilaudid, hydromorphone, Opana, OxyContin, acetaminophen, gabapentin, benzos, Roxicodone

**Suboxone**

Buprenorphine, Subutex, agonist, clonidine, tramadol, hydrocodone, Dilaudid, Vicodin, Sublocade, Percocet, phenibut, Klonopin, Valium

**Heroin**

Dope, opium, opiates, crack, diacetylmorphine, China white, codeine, acetaminophen

**Drug nerds**

Methadone, alkaloids, mitragynine, benzos, poppy, buprenorphine, antagonist, gabapentin, naloxone, amphetamine, hydrocodone

**Research chemicals**

Benzos, psychoactive, psychedelic, kratom, pyrovalerone, quaalude, oxycodone, morphine, Xanax, tramadol, cocaine, methadone, ketamine, gabapentin, amphetamine, hydromorphone

## Methods

### Overview

In this section, we build upon the previous data collected to create Bidirectional Encoder Representations From Transformers (BERT)–based sentiment, emotion, and SUD models. We leveraged those models to predict if a post could be classified as SUD present (SUDP) or SUD absent (SUDA) while considering the history of the post. We applied stratified random sampling [46] to identify a sample population that best represents all the features of interest and ensures that every data subgroup is represented, thus avoiding potential bias in the several data sets we collected for this study.

### Sentiment Analysis and Sentiment BERT Model

**Figure 5.**
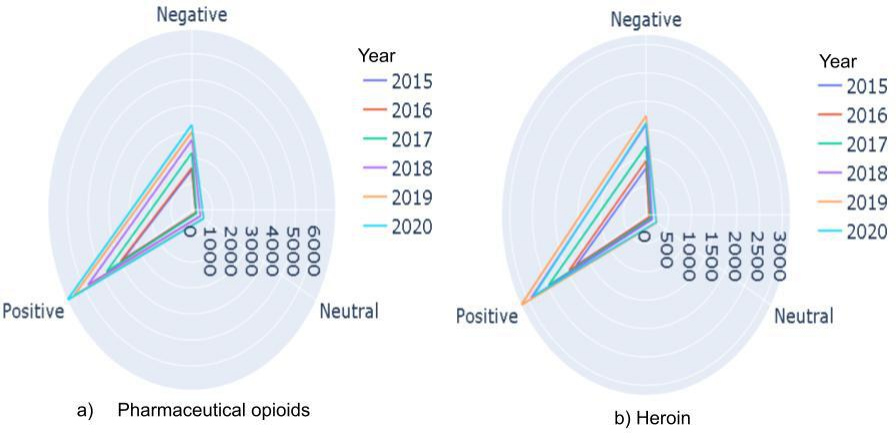
Dark web to social media sentiment analysis module comparison of the drug categories pharmaceutical opioids and heroin by sentiments.

We classified subreddit posts as positive, negative, and neutral for the sentiment analysis. We implemented Valence Aware Dictionary for Sentiment Reasoning (VADER) [47] to generate sentiment for each subreddit post in the SUDS to consider both the polarity and intensity of each sentiment. VADER uses a lexicon of words with human-annotated sentiment polarity scores like SentiWordNet, AFINN, and the National Research Council Canada Word-Emotion Association Lexicon. We chose VADER as it is a rule-based sentiment analysis tool that is specifically attuned to sentiments expressed in social media, and it uses a combination of sentiment-related words, emoticons, and syntax to produce a sentiment score for a given text. Following the individual scoring of each word, the ultimate sentiment is determined by performing a pooling procedure, such as averaging all the sentiments. This data set is split into train, dev, and test sets (75:5:20). The generated training set is used to train state-of-the-art deep learning algorithms like CNN, LSTM, and BERT. The highest *F*_1_-score achieved was 82.36 with the BERT model. We trained the sentiment BERT model on this training data for later use. We report the statistics of sentiment labels for subreddit posts obtained from sampling 800 random data points from each drug category in Table 4. The comparison of the drug categories of pharmaceutical opioids and heroin by the top three sentiments—positive, negative, and neutral—for the period between 2015 and 2020 is presented in Figure 5, which shows the temporal variation in sentiment for each drug.

**Table 4.** Dark web to social media sentiment and emotion analysis module-sentiment stats (number of posts) after sampling 800 random points for each drug category identified from 6 subreddits and the top emotions identified for each drug from Twitter.

Drug	Positive, n	Negative, n	Neutral, n	Top 3 emotions
Opium	481	218	101	Sadness, love, joy
Oxycodone	460	245	95	Sadness, fear, thankfulness
Kratom	459	231	110	Love, sadness, fear
Fentanyl	467	274	59	Sadness, love, fear/thankfulness
Heroin	455	255	90	Sadness, joy, thankfulness
Synthetic heroin	500	240	60	Sadness, fear, thankfulness
Pharmaceutical fentanyl	570	197	33	Sadness, love, joy/thankfulness
Nonpharmaceutical fentanyl	502	264	34	Sadness, love, thankfulness

### Emotion Analysis and Emotion BERT Model

**Figure 6.**
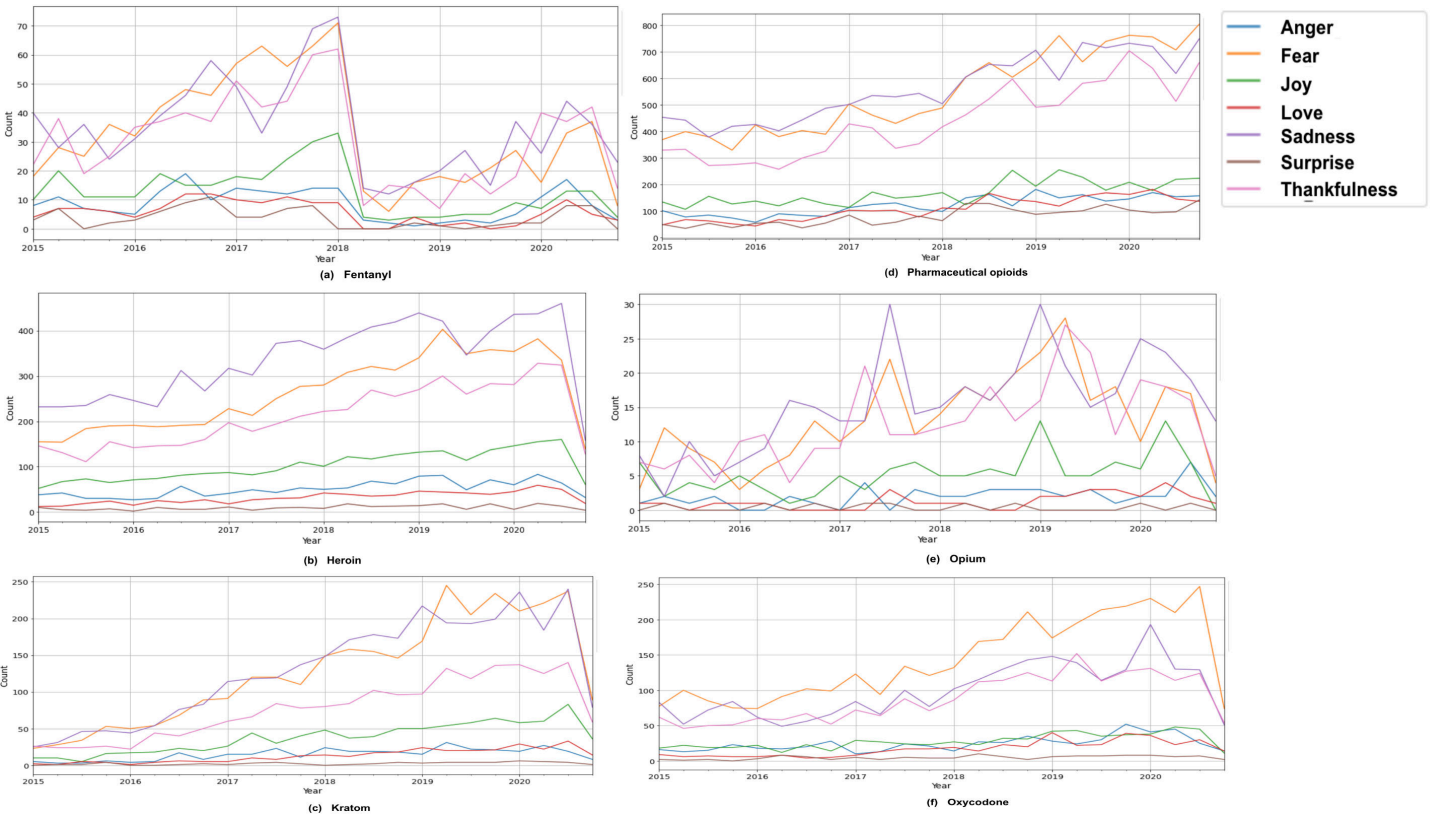
Dark web to social media emotion analysis module comparison of the 8 major drug categories across 7 emotions: joy, sadness, anger, love, fear, thankfulness, and surprise.

We did not choose to work on subreddit data for emotion analysis as we did not have self-tagged emotions in posts on the subreddits. Therefore, we chose to crawl Twitter for the emotion analysis, where emotions are presented as hashtags. We limited our crawl to 7 emotions, as stated by Wang et al [48]. The tweets are assigned a class label corresponding to the emotion hashtag they are associated with. Furthermore, we removed any URLs or usernames that could potentially contain sensitive information. For generating emotion labels for drug-related tweets, we implemented an inductive transfer learning approach with BERT [49]. For this task, we extracted 61,000 posts as labeled training data by crawling tweets with each emotion hashtag: joy, sadness, anger, love, fear, thankfulness, and surprise. We split this data set into train, dev, and test sets (75:5:20). We trained Emotion BERT, which is a BERT-based model for 10 epochs using a learning rate of 1e-5, on a batch size of 32 of the labeled data and on Emonet, a corpus of around 790,000 tweets [50], to generate the emotion labels for subreddit posts in the SUDS. We report the statistics of emotion labels for subreddit posts obtained from sampling 800 random data points from each drug category reported in Table 4. The comparison for all drugs across the 7 emotions: joy, sadness, anger, love, fear, thankfulness, and surprise is shown in Figure 6.

### Substance Use Disorder Data Set

We focused on building and interpreting a predictive model based on these exploratory results to identify posts where SUD is present or absent. We formulated this problem as a binary classification task to predict a label for a post at a particular time. Each post was associated with a drug name, historical posts, time, emotion, and sentiment. We then prepared our training data set to generate SUDP and SUDA labels for the SUDS. We made use of high-quality addiction-labeled data from Lokala et al’s [51] work on social media data for exploring the association between drug and mental health symptoms. Lokala et al [51] created a labor-intensive high-quality corpus of 9888 tweets manually annotated by domain experts and substance use epidemiologists with experience in interventions, treatment, and addiction research. We trained a transfer learning BERT model for 10 epochs using a learning rate of 1e-5 and batch size of 32 on this labeled data to generate the SUDP and SUDA labels for posts in the SUDS. We also examined manual interannotator agreement (κ=0.74) among three domain experts for SUDP and SUDA labels of 300 posts to validate the annotations. The manual annotations were evaluated in the same way as automated labels, and our macro–*F*_1_-score measure against the ground truth was 0.71. The results for transfer learning using BERT are reported in Table 5.

**Table 5.** Validation results for emotion Bidirectional Encoder Representations From Transformers (BERT) and substance use disorder (SUD) BERT models through the transfer learning approach. The trained model was then used to obtain the emotion labels SUD present and SUD absent for the posts in the Substance Use Disorder Corpus.

Transfer learning BERT model	Precision	Recall	*F*_1_-score
Emotion BERT	80.12	82.29	81.19
SUD BERT	81.28	83.65	82.44

### Temporal Predictive Model of Posts to Detect SUDP or SUDA

**Figure 7.**
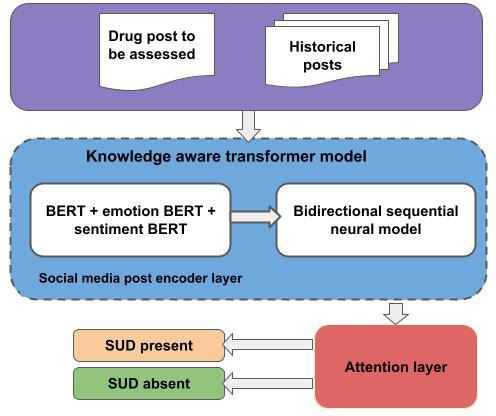
Knowledge-aware time series analysis computation model: a dark web to social media computational module. BERT: Bidirectional Encoder Representation From Transformers; SUD: substance use disorder.

We built our domain-specific sentiment BERT model to serve as a sentiment feature extractor over historical tweets and a domain-specific emotion BERT model as an emotion feature extractor for historical tweets. We built fine-tuned BERT models as they can capture a better sense of sentiment, emotions, social media jargon, and slang terms [52]. We contributed to the knowledge-aware time series analysis computation model to predict SUDP and SUDA for a post as shown in Figure 7. We presented the SUD detection as a binary classification model with SUDP and SUDA as labels. We focused on building and interpreting a predictive model based on these exploratory results to identify posts where SUD was present or absent. Each post was associated with the drug, historical posts, time, emotion, and sentiment in the SUDS.

For a post, the concepts, slang terms, and synonyms related to drug entities are masked using the DAO, which forms the knowledge component of the model. We used BERT to encode the language representation, as BERT can produce more thorough representations of linguistic elements in social media data [52], and we averaged the vector outputs for all tokens in each post at the final layer. To extract emotional language features from posts, we used our emotion BERT model that takes the historical posts and obtains the 768-dimensional emotion vector of each historical post. To extract sentiment language features from posts, we used our sentiment BERT model that takes the historical posts and obtains the 768-dimensional sentiment vector of each historical post. We then have the encoding representing the sentiment and emotion spectrum. Sequential models such as recurrent neural network and LSTM models are apt ways to encode representations that learn from a sequence of a user’s historical tweets due to the sequential nature of a social media post history. We then passed the historic posts through a bidirectional LSTM + attention layer concatenated with the post to be assessed. We then fed the extracted features from the attention layer to a dense layer with the rectified linear unit to get the prediction vector. Finally, we used the softmax function to output the probability that the post is labeled as SUDP or SUDA.

For experiments, we split the data set into a 75:5:20 ratio for the train, development, and test sets, respectively. We fine-tuned the hyperparameters using the development set. Each model was trained for 10 epochs with a learning rate of 1e-5 and a batch size of 64. We used cross-entropy loss and Adam [53] for the optimization. For regularization, we used dropout [54] with a probability of 0.2. We got the best performance with the bidirectional LSTM model with the attention layer as it captured context over a longer span considering the bidirectional context of a word. For all models, we report the recall, precision, and *F*_1_-score. We interpreted the higher performance gains of our model in the Results section through an ablation study.

### Performance Comparisons

We compared the performance of these state-of-the-art methods through replications of the architectures and representations presented in prior works on similar tasks on social media.

- Logistic regression [55]: We implemented a logistic regression classifier that uses the part of speech (POS) and TF-IDF as language feature representations.
- Random forest [56]: We implemented a random forest model with features like Linguistic Inquiry and Word Count (LIWC), POS, and TF-IDF.
- History-aware recurrent neural network (H-RNN) [57]: We deployed H-RNN that encodes input using fine-tuned fast text embeddings. Historical posts are passed sequentially through the model and concatenated with the post to be assessed. The sigmoid activation was selected for the hidden LSTM layer, which is fully connected to both the input and output layers.
- History-aware LSTM (H-LSTM) [58]: We replicated H-LSTM that used BERT embeddings for encoding historic posts given to an attention-based LSTM layer, allowing the model to choose whether to focus more or less on each post to reflect user representation, which was finally be fed to a fully connected layer with a sigmoid activation function to get the prediction.

### Ethical Considerations

We applied our model to study how historic emotion and sentiment of a drug impacts social media conversation dynamics related to substance use. An important aspect that we need to consider while working with addiction-related issues is to respect the users’ privacy and adhere to good ethical practices adopted by previous research [59,60]. Therefore, similar to Matthews et al [61], we censored sensitive information such as usernames, personal information, platform identifiers, and URLs that might be directly linked to the user’s identity from the collected posts. All examples used in this paper are anonymized and deidentified for user privacy [62]. We also adopted the proposed guidelines for the treatment of names and online pseudonyms in posts gathered from social media [63]. In this work, we study substance use in subreddit groups in the form of textual interactions. The expressed addiction intent may differ from the intent perceived or experienced by the person. However, obtaining perceived intent from social media is challenging and involves ethical risks. Before behavioral health intervention apps based on social media data can be used in real-world settings, difficulties with potential biases and user privacy must first be resolved, along with establishing suitable regulations and boundaries in this domain. We can adopt the approach used in this work to develop the data set with more human supervision, and we acknowledge that the data may be prone to demographic-, annotator-, and platform-specific biases [64,65]. We also acknowledge that this study does not make any clinical diagnosis or treatment suggestions directly. However, its findings can still be highly valuable for informing clinical practice and interventions for SUD in the identification of risk factors. Even if the study does not prescribe specific treatments, its findings can inform the development of novel intervention strategies or the optimization of existing ones. For example, as our paper highlights certain sentiment and emotion patterns associated with SUD, clinicians can incorporate this knowledge into their approaches. This research also provides further understanding of the factors contributing to the onset of SUD, which can help in designing prevention programs aimed toward at-risk populations, such as individuals with mental health disorders. Research findings can inform policy makers about the effectiveness of current strategies and the need for adjustments in regulations, health care policies, or resource allocation for addressing SUD at a broader level.

This study was submitted for review by the institutional review board at the Wright State University and has been determined to meet federal exemption criteria: 45 CFR 46.101(b)(4).

## Results

### Overview

Of all opioid listings in the eDark data set, 4.2% are related to novel synthetic opioids, and heroin was identified in 57.8% of all opioid-related listings. When comparing the average monthly ad volume for fentanyl, fentanyl analogs, and other nonpharmaceutical drugs, data indicates a rise in the availability of items containing fentanyl. The listings for pharmaceutical and nonpharmaceutical fentanyl and analogs made up 1.9% of all opioid-related listings, which is 48.6% of the unique synthetic opioid-related ads. The most frequent type of novel synthetic opioid, which was synthetic heroin, was offered for sale at an average of 1.6 kg at each time point of data collection during the study period. Furanylfentanyl was the fentanyl analog that was promoted the most with an average of 3.6 kg being offered for sale at each data point. Carfentanil, a highly strong fentanyl analog, was typically available for purchase for 489.6 grams on average. Newer synthetic opioids (eg, U-48800, U-4TDP) kept replacing the nonpharmaceutical synthetic opioids (eg, W-18, MT-45, AH-7921, U-47700) in the listings found on marketplaces.

From the exploratory analysis on the SUDS, kratom, heroin, fentanyl, morphine, cocaine, methadone, Suboxone, and oxycodone were the most commonly discussed drugs across 6 subreddits. In Textbox 2, considering the Research Chemicals subreddit, for example, it is interesting to find that more posts talk about pyrovalerone, a psychoactive drug with stimulant effects. Another term found is “Quaalude,” a brand name for “Methaqualone,” a sedative and hypnotic medication. The Research Chemicals subreddit mostly discusses psychoactive and psychedelic drugs, while DrugNerds discussed alkaloids [66]. Interestingly, DrugNerds talked about naloxone, which can treat opioid overdose. Dope is a slang term for heroin and was identified in the Heroin subreddit. Several brand names of medications for anxiety, pain, seizures, insomnia, and sedatives are discussed in the Suboxone subreddit. Gabapentin is the typical seizure and pain medication discussed among most of the subreddits. The Opiates Recovery subreddit is more about withdrawal symptoms and mental health disorders, for example, “cold turkey.” The term “cold turkey” used in the context of substance use is quitting a substance abruptly, which carries significant risks if the drug you are discontinuing is a benzodiazepine or opiate [67,68]. The results show that we can derive and analyze slang terms, brand names, novel drugs, mental health symptoms, and medications from social media. From the results in Table 4, the highest positive sentiment was found in pharmaceutical fentanyl, the highest negative sentiment in fentanyl, and the highest neutral opinion in kratom. The emotion “Love” was most detected in posts related to kratom as people use it for self-medication. Table 6 presents the medians of metrics for different embeddings and architectures obtained over 20 runs. The baseline models we compared our model to were logistic regression, random forest, H-RNN, and H-LSTM with varied language representations like POS and TF-IDF. We also extracted LIWC features from posts to pass through a predictive model instead of BERT encoding. Under identical circumstances, we empirically discovered that BERT outperformed LIWC considerably (*P*=.03). We presented model interpretability and significance as an ablation study.

**Table 6.** Median of metrics for different embeddings and architectures that were obtained over 20 runs.

Representation	Model	Macro–*F*_1_-score	Precision	Recall
POS^a^ + TF-IDF^b^	Logistic regression	52.72	47.67	50.31
Linguistic Inquiry and Word Count + POS + TF-IDF	Random forest	55.84	54.63	56.78
Fast text	History-aware recurrent neural network	74.47	69.48	76.17
BERT^c^	History-aware long short-term memory	76.85	70.56	77.61
Knowledge-aware BERT	Knowledge- and history-aware bidirectional long short-term memory + attention	*82.12^d^*	*78.34*	*83.58*

a POS: part of speech.

b TF-IDF: term frequency–inverse document frequency.

c BERT: Bidirectional Encoder Representations From Transformers.

d Italics denote that the result is significantly better than history-aware recurrent neural network (*P*=.04).

We used the Wilcoxon signed rank test [69] to compare the emotional expression in posts and comments between those with and without substance use to assess statistical significance. There was a significant correlation between the emotion displayed in posts labeled SUDP (*P*<.001) and the posts labeled as SUDA. We next conducted an ablation study where we removed one component from our model and assessed the performance to analyze the prime components in our methodology. We concatenated the substance use post encoding e_i_^(S)^ and emotion post encoding e_i_^(E)^, and used the resulting representation as the input to the linear layer to exclude the attention component from the model. We trained our encoders with raw data that was directly collected from social media to remove the entity-masking component. Additionally, we trained our model by merely training the classifier and excluding the post history from the model. In Table 7, we report the findings for the SUD prediction task for posts. Entity masking, which considerably improves the SUD identification task (+3.73 precision, +3.43 recall), is where we saw gains. The Wilcoxon signed rank test demonstrated that contextualized representation is very desirable for the SUD identification task in this study since it performs better than the model without entity masking (*P*=.04). Additionally, adding the history of the post significantly boosted the performance where we saw our highest increase (+4.62 precision). Attention also increased the model’s precision by 1.85% and recall by 3.24%, meaning that every feature of the model affects how well it performs this task. Below, we discuss the examples of SUD and the result error analysis.

**Table 7.** Ablation study: median of metrics over 10 different runs.

Model	*F*_1_-score, median (change from proposed model)	Precision, median (change from proposed model)	Recall, median (change from proposed model)
Proposed model (entity masking + attention + history of post)	*82.12* ^*a*^	*78.34*	*83.58*
Minus attention	78.98 (3.14↓)	76.49 (1.85↓)	80.34 (3.24↓)
Minus entity masking	78.50 (3.62↓)	74.61 (3.73↓)	80.15 (3.43↓)
Minus history of post	77.58 (4.54↓)	73.72(4.62↓)	79.12 (4.46↓)

a Italics denote best performance.

### Error Analysis

We analyzed the sources of errors and discuss the predictions made by our models in Table 8 in three scenarios.

1. Polydrug use with variable emotions: For post 1, examining the post where multiple drugs coexist along with emotion variability in the history associated with other drugs, for example, mixing depressants and stimulants or mixing medications with opioids, our model was not able to predict correctly, for example, when substance A might not often co-occur with substance B in the history.
2. Post-level ambiguity: For post 2, our model was able to predict SUD by examining the post even if it was too ambiguous to assess given that the user has a clear SUDP label in the past and was undergoing the healing process, with emotion intensity for the historical posts like increased sadness-related emotions.
3. Sarcasm detection: For post 3, even if it does not contain any clear SUDP/SUDA label, sarcasm identified in the post, with a history of ambiguous posts, presents difficulty in identifying SUD, which makes it an interesting natural language understanding problem and explains the task’s complexity, revealing paths for future work.

**Table 8.** Examples showing major errors made by our proposed approach.

Post/comment	Error type	Actual	Predicted
1. “Imagine a combo. Just got stoned with 12 grams of kratom and 15 g of **** with 40 mg of ***** AMA lol, ended up projectile vomiting, went to sleep feeling fearful and woke up feeling pretty joyful but little shitty, so i had glass of ACV, all is well...”	Polydrug use	SUDP^a^	SUDA^b^
2. “The improved memory isn’t dependent on the supplement. I can take the same dose of Nal****one to treat Opioid Use Disorder for 3 mo and not feel a thing. That tells me that it helped me heal, and there’s not much more for it to do.”	Post-level ambiguity	Ambiguous	SUDP
3. “why SPEAK the UNSPOKEN? Dangerously F***ing disaster. Mixing *** (a long-term damaging drug, with an ability to make other drugs dangerously stronger) with Xanax (addictive,overdoseable drug) and ***** (an opiate that can and likely will kill you) is a recipe for life after ***”	Sarcasm detection	Sarcastic	SUDA

a SUDP: substance use disorder present.

b SUDA: substance use disorder absent.

## Discussion

### Principal Findings

Crawling cryptomarkets poses a significant challenge when applied to data science and machine learning to study the opioid epidemic due to the restricted crawling process [1,43,70]. However, we make our proposed data set available to the research community for further analysis. To identify the best strategies to reduce opioid misuse, a better understanding of cryptomarket drug sales that impact consumption and how it reflects social media discussions is needed [71]. We limited this study to 8 broad drug categories due to the availability and abundance of related posts on the dark web; we hope to refine further and expand our categories for future work. Further, we have identified processes for future research. We plan to expand this work to extract mental health symptoms from the drug-related social media data to connect the association between drugs and mental health problems, for example, the association between cannabis and depression [72,73]. We also plan to build an opioid drug social media knowledge graph (ODSM-KG) with all the diverse data points (drug, sentiment, emotion, mental health symptom, and location) and compare it against the state-of-the-art “Knowledge Graph based Approach For Exploring The U.S. Opioid Epidemic” [71]. Potential areas of application would be identifying risk factors regarding addiction and mental health from subreddit data [74], and identifying drug trends based on location with a possible opioid epidemic prediction. To identify the approaches for mitigating the misuse of opioids, it is imperative to study consumption patterns at the national and regional levels, the influences of the pharmaceutical industry, and the sociopolitical determinants that affect consumption. For our ODSM-KG, we aim to create a web-based tool that will allow for the depiction of historical patterns and enable comparisons between opioids, time periods, and areas within the United States. Given that our ODSM-KG was primarily based on data, we aim to enhance its accuracy and effectiveness by seeking guidance from a subject matter domain expert. This will enable us to customize it for unique scenarios and cater to the needs of specific users. We would also like to include Drug Enforcement Agency drug seizures in our preliminary data collection process to be aware of related social media discussions.

### Global Relevance

SUD represents a substantial public health challenge worldwide, with far-reaching implications for individuals, families, and communities across diverse cultural and geographic contexts. Despite efforts by governments and health care organizations, the detection and intervention of SUDs remain complex and multifaceted, often hindered by stigma, limited resources, and barriers to early identification. We aim to examine the global relevance of detecting SUD through social media analysis, highlighting the potential of innovative approaches proposed in this study. Our study explores the potential of leveraging social media data, including content sourced from the dark web, to detect indicators of SUD and inform targeted interventions. By applying both temporal dynamics and domain-specific knowledge, we can extend this research to enhance the accuracy and effectiveness of SUD detection on a global scale. Collaborative efforts involving researchers, policy makers, and health care professionals from diverse regions can facilitate the adaptation and refinement of our approach to suit different cultural contexts and population groups. By harnessing the approaches implemented in this paper regarding temporal social media analysis and SUD-associated mental health disorders, researchers from other countries can adapt this approach to address these challenges associated with SUD and improve health outcomes for populations worldwide.

## Abbreviations

BERT: Bidirectional Encoder Representations From Transformers
CNN: convolutional neural network
DAO: drug abuse ontology
H-LSTM: history-aware long short-term memory
H-RNN: history-aware recurrent neural network
LIWC: Linguistic Inquiry and Word Count
LSTM: long short-term memory
NER: named-entity recognition
ODSM-KG: opioid drug social media knowledge graph
POS: part of speech
PREDOSE: Prescription Drug Abuse Online Surveillance and Epidemiology
SUD: substance use disorder
SUDA: substance use disorder absent
SUDP: substance use disorder present
SUDS: Substance Use Disorder Corpus
TF-IDF: term frequency–inverse document frequency
VADER: Valence Aware Dictionary for Sentiment Reasoning
